# Effects of Ginger* (Zingiber officinale Roscoe)* on Type 2 Diabetes Mellitus and Components of the Metabolic Syndrome: A Systematic Review and Meta-Analysis of Randomized Controlled Trials

**DOI:** 10.1155/2018/5692962

**Published:** 2018-01-09

**Authors:** Jie Zhu, Hao Chen, Zhixiu Song, Xudong Wang, Zhenshuang Sun

**Affiliations:** ^1^The Second Clinical College, Nanjing University of Chinese Medicine, Nanjing, Jiangsu 210023, China; ^2^Henan Provincial People's Hospital, Zhengzhou, Henan Province 450003, China

## Abstract

**Objective:**

This article aims to assess the effects of ginger* (Zingiber officinale Roscoe)* on type 2 diabetes mellitus (T2DM) and/or components of the metabolic syndrome (MetS).

**Methods:**

Electronic literature was searched in PubMed, Embase, the Cochrane Library, Chinese Biomedical Database, China National Knowledge Infrastructure, and Wanfang Database from inception of the database to May 19, 2017, and supplemented by browsing reference lists of potentially eligible articles. Randomized controlled trials on research subjects were included. Data were extracted as a mean difference (MD) and 95% confidence interval (CI). Subgroup analysis of fasting blood glucose (FBG) was performed.

**Results:**

10 studies met the inclusion criteria with a total of 490 individuals. Ginger showed a significant beneficial effect in glucose control and insulin sensitivity. The pooled weighted MD of glycosylated hemoglobin (HbA1c) was −1.00, (95% CI: −1.56, −0.44; *P* < 0.001). Subgroup analysis revealed that ginger obviously reduced FBG in T2DM patients (−21.24; 95% CI: −33.21, −9.26; *P* < 0.001). Meanwhile, the significant effects of improvement of lipid profile were observed. Most analyses were not statistically heterogeneous.

**Conclusion:**

Based on the negligible side effects and obvious ameliorative effects on glucose control, insulin sensitivity, and lipid profile, ginger may be a promising adjuvant therapy for T2DM and MetS.

## 1. Introduction

With the changes of lifestyle and living environment, diabetes mellitus is spreading around the world at an alarming rate. As indicated by the data given by International Diabetes Federation (IDF), the number of diabetic patients had increased to 415 million in 2015 and 10% adults will suffer from diabetes in 2040 [1]. Meanwhile, metabolic syndrome (MetS), an important risk factor for diabetes, increased approximately fivefold the risk of type 2 diabetes mellitus (T2DM), and its incidence is as high as one-quarter of the world's adult population [2]. The diseases seriously threatened the public health [3]. Patients with T2DM or MetS share common characteristics of raised blood sugar, decreased insulin sensitivity, obesity, dyslipidemia, and hypertension, which often appear simultaneously rather than alone [4]. Currently, clinical treatment mainly focuses on symptom intervention via pharmacological agents, the side effects of which, especially for long-term usage, however, have resulted in major concerns to the public [5]. Alternative therapy with less side effects is urgently needed.

Ginger* (Zingiber officinale Roscoe)* is herbaceous perennial plant of the family Zingiberaceae, which is used as a spice all over the world for its special pungency and typical aroma [6]. In addition, ginger is one of the most famous medicinal herbs in traditional Chinese Medicine and Indian Ayurvedic System of Medicine for centuries [7]. It is used to treat stomachache, arthritis, nonalcoholic fatty liver disease, primary dysmenorrhea, and nausea caused by pregnancy and chemotherapy [7–11]. Wang et al. [12] indicated that ginger was a promising therapy for T2DM and MetS through multiple targets and pathways. This positive effect may be resulting from its primary bioactive ingredients such as gingerols, shogaols, zingerone, and paradols [13]. However, there was inconsistent voice: Bordia et al. reported that neither blood glucose nor lipid was changed in patients with coronary artery disease after taking 4 g of ginger powder for 3 months [14]. Such discrepancy of results may be attributed to the variation in chemical composition of the ginger products, depending on the preparation method, producing area, or storage condition [15]. Our systematic review aimed to summary the convincing evidence of current studies to clarify the efficacy of ginger on T2DM and components of MetS.

## 2. Methods

This systematic review and meta-analysis were conducted following a predetermined protocol established according to the Cochrane Handbook's recommendations [16] and results were reported in accordance with the Preferred Reporting Items for Systematic Reviews and Meta-Analyses (PRISMA) statement [17]. It was registered in PROSPERO. The registration number is CRD42017069241 (https://www.crd.york.ac.uk/PROSPERO).

### 2.1. Search Strategy

An electronic literature search was carried out by two authors (HC and SZS) independently from inception of the database to May 19, 2017, in the following electronic databases: PubMed, Embase, the Cochrane Library, Chinese Biomedical Database (CBM), China National Knowledge Infrastructure (CNKI), and Wanfang Database. Search terms consisted of thesaurus terms and free terms. In PubMed, sensitivity-maximizing search strategy was used for identifying randomized trials [16]. The search terms were adjusted according to the requirements of the database-specific filters for randomized controlled trials (RCTs). The complete list of search terms for PubMed is available (eTable 1). The search was supplemented with potentially eligible articles by browsing the literature in the reference lists and manual search was conducted through relevant journals in the field of diabetes and MetS.

### 2.2. Inclusion and Exclusion Criteria

In order to estimate the effects of ginger on T2DM and components of MetS, we included studies in which (1) the subjects of the study are suffering from T2DM and/or at least one of components of MetS according to the International Diabetes Federation standards [2]; (2) the intervention is limited to ginger alone; (3) the study is carried out with randomized controlled trial. Exclusion criteria were as follows: (1) nonstandardized diagnosis; (2) the control group being treated with other methods besides the placebo. The longest intervention duration was adopted in studies with more than one end-point. In the case of multiple publications from the same trial, all the data of outcomes of interest were extracted and utilized. Editorials, case reports, and correspondences were excluded.

### 2.3. Study Selection

The study selection was based on the process of PRISMA suggestions [17]. First of all, the duplicate studies were removed; then two reviewers (HC and SZS) independently screened the titles and abstracts of all retrieved literatures according to the inclusion and exclusion criteria, and a list of potentially eligible articles was formed. The next step was that two reviewers (HC and SZS) considered full text of these potential studies for further selection. Disagreements were resolved by consensus. Meanwhile, a third reviewer (JZ) was available for mediation throughout the whole process. If literature was not available in the publication and could not be excluded yet, we contacted the corresponding authors for potential articles to confirm eligibility. Authors of studies were contacted up to two times within 3 weeks.

### 2.4. Data Extraction

Two reviewers (HC and SZS) conducted data extraction separately according to a predetermined standardized form. The controversies were settled through consultation with a third party (JZ). For each included article, we extracted information as follows: (1) first author's name, publication year and country, sample size, population characteristics, intervention methods and doses, follow-up duration, and study design; (2) outcomes of interest: serum triglyceride (TG), serum total cholesterol (TC), high density lipoprotein-cholesterol (HDL-c), low density lipoprotein-cholesterol (LDL-c), fasting blood glucose (FBG), glycosylated hemoglobin (HbA1c), homeostasis model assessment-insulin resistance index (HOMA-IR), and body mass index (BMI). All the values scales were unified before they were combined.

### 2.5. Assessment of Risk of Bias

We assessed risk of bias for each included study using the Cochrane Collaboration “Risk of bias” assessment tool. The risk of bias evaluation standards of the Cochrane Handbook was presented in the following specific domains, namely, selection bias (random sequence generation and allocation concealment), performance bias (blinding of participants and personnel), detection bias (blinding of outcome assessment), attrition bias (incomplete outcome data), reporting bias (selective reporting), and other sources of bias [18]. Two reviewers (HC and SZS) assessed each item with “yes,” “no,” or “unclear” for “low risk,” “high risk,” or “unclear risk” of bias correspondingly. Disagreement in the assessment was resolved through consultation with the third party (JZ). All the assessments were conducted with RevMan software (V.5.3.5).

### 2.6. Data Synthesis and Analysis

The Review Manager 5.3.5 tool, provided by the Cochrane Collaboration, was used to analyze data and generate forest plots. A fixed-effect model was used when there was no statistical heterogeneity; otherwise, a random-effect model was applied [19]. Heterogeneity was assessed using the degree of freedom *P* value and *I*^2^-test statistic. Value of *P* < 0.10 or value of *I*^2^ > 50% suggested significant heterogeneity and the result of the random-effect model was reported [20]. In addition, meta-analysis was performed when data of outcomes of interest were available from at least two studies. The weighted mean difference (WMD) with 95% confidence interval (CI) was calculated for continuous variables. Significance level was set at *P* ≤ 0.05. Subgroup analysis was conducted according to the types of the disease when potential differences among studies included were observed in the quantitative synthesis.

## 3. Results

**Figure 1:**
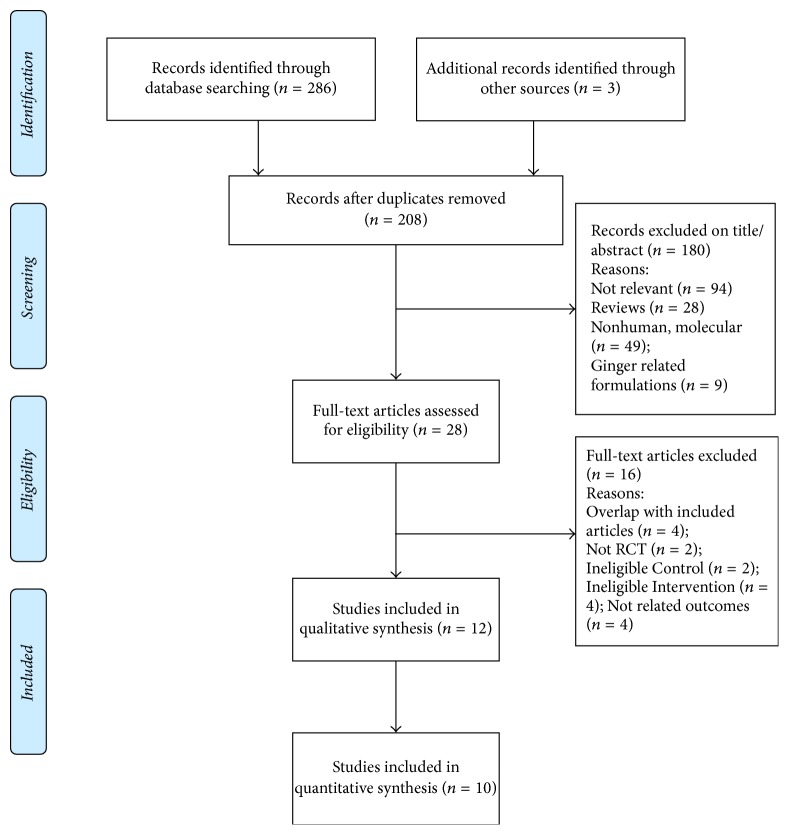
Search process flowchart.

A total of 289 records were identified for the initial search, and 12 articles met the inclusion criteria, which were included in systematic review [21–32]. Since there were no results of outcomes of interest in two records [22, 30], 10 articles were finally included in meta-analysis [21, 23–29, 31, 32]. The details of the selection process and the reasons for exclusion of studies are summarized in Figure 1.

### 3.1. General Study Characteristics

Primary individual study characteristics are summarized in Table 1. It turned out that 11 studies were conducted in Iran and 1 was in India. The total sample size covered 490 patients. Of the total 12 trials included, 5 trials [22–26] focused on T2DM subjects who mostly took oral hypoglycemic drugs, but no one used insulin. Four trials [27–30] focused on obesity, 2 articles [31, 32] on continuous ambulatory peritoneal dialysis (CAPD) subjects with hyperlipidemia or hyperglycemia, and one trial [21] on hyperlipidemia. During the treatment, patients were treated with oral administration of 1–3 g capsules or tablets of ginger rhizome per day. The capsules or tablets were taken once [26, 28, 29], twice [23, 24], three times [21, 22, 25], or four times [27, 30–32] daily and after meals. The follow-up duration varied from 30 days to 3 months. Experiment participants were carefully instructed to avoid changes of their physical activity routines or dietary patterns during the study period, while two articles did not address specific details [21, 30].

### 3.2. Quality Assessment

**Figure 2:**
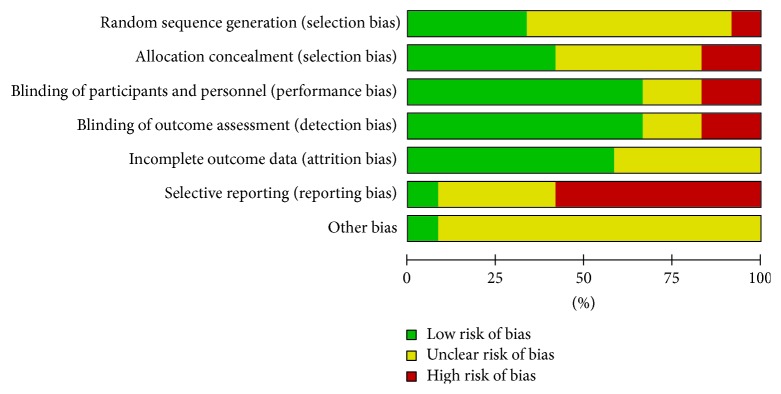
Risk of bias item presented as percentages across all included studies.

The evaluation on each risk of bias item across all included studies was conducted (eTable 2). Although the included studies were carried out with RCTs, the method of randomization was declared only in four studies [25, 26, 28, 29], and allocation concealment in five studies. In addition, seven articles were at high risk in terms of selective reporting because of multiple reports from the same study with different outcomes of interest [28, 29, 31, 32] and incomplete data of outcomes [22, 27, 30]. The overall evaluation graph presented as percentages is formed from RevMan tool (Figure 2).

### 3.3. Outcomes

#### 3.3.1. Effects of Ginger on Glucose Control and Insulin Sensitivity

**Figure 3:**
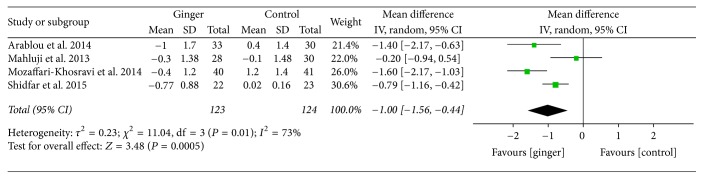
Meta-analysis of the effect of ginger on HbA1c compared with that of placebo.

**Figure 4:**
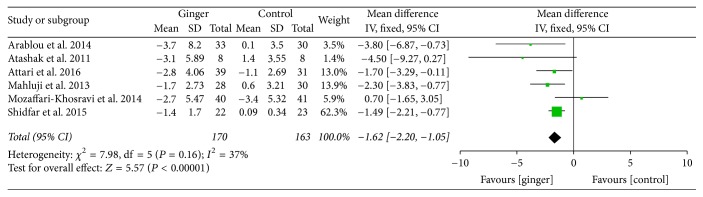
Meta-analysis of the effect of ginger on fasting insulin compared with that of placebo.

**Figure 5:**
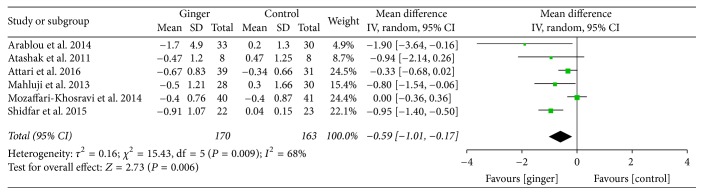
Meta-analysis of the effect of ginger on HOMA-IR compared with that of placebo.

Ginger powder could significantly improve blood glucose and insulin sensitivity compared with the placebo. Mean changes of HbA1c (% total) were available in 4 studies [23–26]. WMD and 95% CI of HbA1c (−1.00; 95% CI: −1.56, −0.44; *P* < 0.001) indicated significant change after ginger administration versus placebo (Figure 3). The mean changes in fasting insulin concentrations (*μ*IU/ml) were measured in 6 studies [23–27, 29] and significant reduction was observed between ginger groups and the control groups (−1.62; 95% CI: −2.20, −1.05; *P* < 0.001) (Figure 4). In addition, 6 studies [23–27, 29] focused on insulin sensitivity which was assessed by HOMA-IR index. The pooled net change (−0.59; 95% CI: −1.01, −0.17; *P* < 0.01) showed that the insulin sensitivity was obviously enhanced (Figure 5).

#### 3.3.2. Effects on MetS Profile

**Figure 6:**
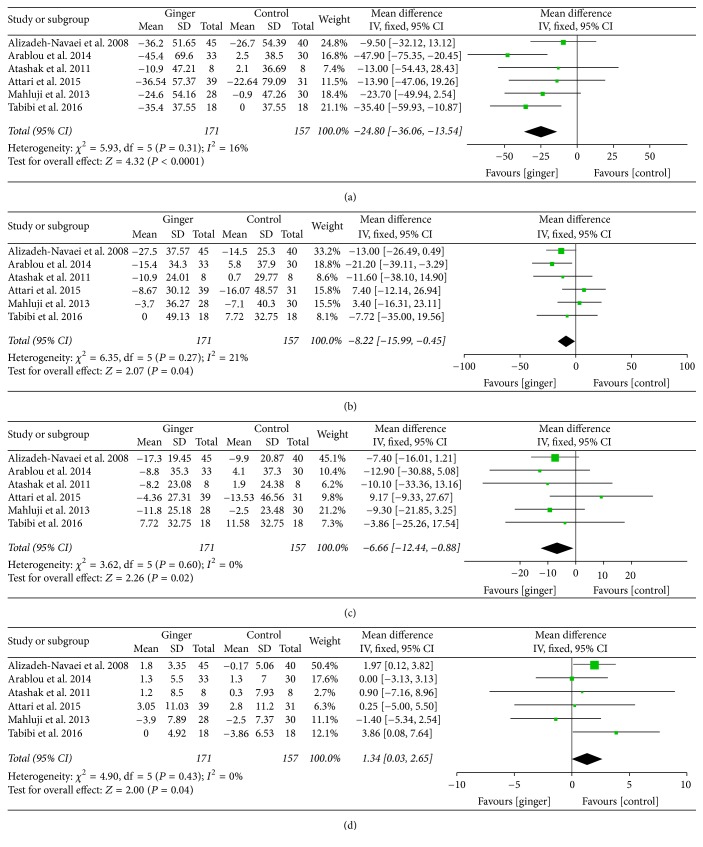
Meta-analysis of the effect of ginger on lipid profile compared with that of placebo: (a) for TG, (b) for TC, (c) for LDL-c, and (d) for HDL-c.

MetS profile was evaluated by serum lipid parameter and BMI based on available results of included studied. The mean differences of TG, TC, LDL-c, and HDL-c concentration were pooled from 6 studies [21, 23, 24, 27, 28, 32]. WMD and 95% CI of TG (−24.80; 95% CI: −36.06, −13.54; *P* < 0.001), TC (−8.22; 95% CI: −15.99, −0.45; *P* < 0.05), and LDL-c (−6.66; 95% CI: −12.44, −0.88; *P* < 0.05) indicated that there was significant difference between the ginger and control groups. Meanwhile, a significant increase was observed in HDL-c (1.34; 95% CI: 0.03, 2.65; *P* < 0.05) (Figure 6).

**Figure 7:**
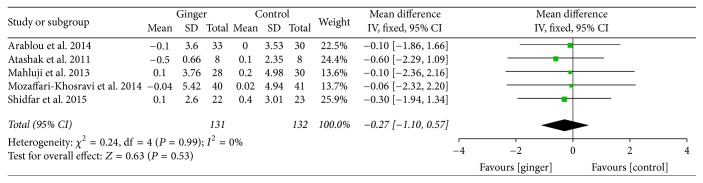
Meta-analysis of the effect of ginger on BMI compared with that of placebo.

Mean changes of body mass index (BMI) were available in 5 studies [23–27]. From the perspective of the variation trend of BMI, ginger could lower BMI, but the change was so slight that it showed no statistical significance (−0.27; 95% CI: −1.10, 0.57; *P* = 0.53) (Figure 7).

### 3.4. Sensitivity and Subgroup Analysis

Sensitivity analysis revealed that result of the meta-analysis was not affected when deleting any of the included RCTs by one-study remove approach. There was significant heterogeneity in the effects of ginger on FBG, HbA1c, and HOMA-IR but not for other outcomes. These heterogeneities were mostly attributed to the study of Mozaffari-Khosravi et al. [25]. By removing the mentioned study, notable diminution of heterogeneities was observed and statistical significance of pooled effect size was stable as before.

**Figure 8:**
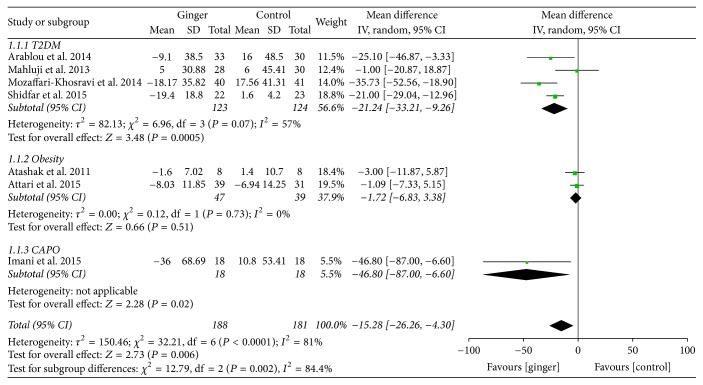
Subgroup analysis of the effect of ginger on FBG (mg/dl) compared with that of placebo.

A subgroup analysis was implemented in FBG according to the type of the disease due to the considerable heterogeneity for the 7 RCTs included. FBG in subjects with T2DM changed significantly between the groups (−21.24; 95% CI: −33.21, −9.26; *P* < 0.001) and CAPD (−46.80; 95% CI: −87.00, −6.60; *P* < 0.05), whose baseline FBG was obviously higher than normal value. However, no significant difference was found in obese subjects (−1.72; 95% CI: −6.83, 3.38; *P* = 0.51) whose baseline FBG was within normal condition [27, 28] (Figure 8). Inconsistent results may be caused by the differences of the baseline blood sugar and the severity of the disease.

### 3.5. Adverse Events

The study showed that prolonged daily intake of ginger powder preparations would lead neither to serious adverse effects nor to complications which normally occurred in the administration of hypoglycemic or hypolipidemic drugs. Of 12 studies included in current systematic review, only one trial reported slight adverse event. In this RCT, Arablou et al. [23] reported only one case with symptom of heartburn in the ginger group during the early stage of the experiment, while other patients had no discomforts. In a word, ginger was generally considered not only as a safe medicinal plant based on studies included but also as a food in the FDA's “generally regarded as safe” list [33].

### 3.6. Publication Bias

Publication bias was evaluated by the funnel plots (eFigures 1–3). No significant publication bias was noticed in the current meta-analysis in FBG, HbA1c, HOMA-IR, TC, LDL-c, and BMI. However, a mild dissymmetry was observed by visual inspection in fasting insulin, TG, and HDL-c, which indicated that there was slight publication bias. It may be caused by the small number of RCTs included, limited sample sizes, and the majority of positive results of outcomes of interest.

## 4. Discussion

To the best of our knowledge, this is the first article to evaluate the effects of ginger on type 2 diabetes mellitus and components of the metabolic syndrome. Our meta-analysis revealed that ginger could significantly reduce fasting blood glucose and HbA1c. Meanwhile, as indicators of pancreatic function and insulin resistance, fasting insulin and HOMA-IR were significantly improved after ginger consumption. In addition, most of MetS risk factors were ameliorated by ginger preparations. However, there was no significant effect in lowering BMI.

### 4.1. Glucose Control and Insulin Sensitivity

A notable discrepancy was found in the effects of ginger on FBG according to the subgroup analysis. The significant lowering effect of ginger was observed in the subgroups of T2DM and CAPD. The subgroup analysis elucidated that the beneficial effect of ginger (i.e., decreasing fasting blood glucose) may be more prominent in patients with hyperglycemia than without. This finding was consistent with the previous studies which found FBG was significantly decreased and glucose intolerance was markedly improved after ginger administration of diabetic rats [34–36]. The underlying mechanism involved the inhibition of *α*-glucosidase and *α*-amylase which are key enzymes in the digestion and absorption of complex carbohydrate [37]. Besides, 6-Gingerol, a component of ginger, showed potent activity in stimulating glucose metabolism via the AMPKalpha2-mediated AS160-Rab5 pathway and through potentiation of insulin-mediated glucose regulation [38]. 6-Paradol and 6-shogaol, which are the pungent compounds of ginger, also have the same effects [39]. Ginger extraction could also enhance the expression of glucose transporter type 4 (GLUT-4) [40] and increase GLUT-4 to promote glucose uptake in adipocytes and skeletal muscle cells [41]. Furthermore, ginger could reduce fasting insulin level and HOMA-IR. Insulin sensitivity could be enhanced by upregulating adiponectin and peroxisome proliferative activated receptor *γ* (PPAR *γ*) [42] and by ginger constituent interaction with the 5-HT_3_ receptor [43]. Moreover, 6-Gingerol extracted from ginger could protect pancreatic *β*-cells [44]. The reduction in HbA1c further suggested that ginger has long-term blood sugar lowering effect.

### 4.2. Lipid Profile

Although ginger significantly improved dyslipidemia according to our meta-analyses, there was noticeable inconsistency among individual study results. For example, the study of Atashak et al. [27] showed no significant changes of all blood lipid parameters in the obese when taking 1 g of ginger capsules daily for 10 weeks. Four RCTs [24, 27, 28, 32] reported that ginger failed to significantly decrease serum TC. Five studies [21, 23, 27, 28, 32] showed that plasma LDL-c concentration was not lowered in ginger groups versus placebo. Such inconsistency may be caused by difference of type and severity of the disease, preparation method, dose intervention level, and follow-up duration. The antihyperlipidemic effect of ginger was supported by animal studies [45, 46]. The activities of ginger can be ascribed to its acceleration of lipid metabolism by the modulation on the expression of marker enzymes [46] and its downregulation of retinoid-binding protein (RBP) mRNA expression levels in the liver and visceral fat which is lipid metabolism related gene and an important indicator of hyperlipidemia [47]. Meanwhile, ginger inhibits the conversion of excess carbohydrates into TG by regulating the expression of carbohydrate response element-binding protein (ChREBP) [48]. In addition, the lowering effect of ginger on serum cholesterol may be due to the inhibitory effect of cholesterol biosynthesis [49] and the transformation of cholesterol into bile acids by elevating the activity of hepatic cholesterol 7 alpha-hydroxylase [50]. Furthermore, niacin, a nutrient in ginger [51], may be a potential active ingredient in lowering serum triglyceride level, increasing clearance of VLDL, enhancing hepatic uptake of LDL-c, and inhibiting cholesterol synthesis [52, 53].

### 4.3. Body Mass Index

Although there was no significant change in BMI according to the pooled result, the trend of forest plot indicated that ginger may be beneficial to reduce BMI of the obese, which has been reported in many studies [54–56]. Mahmoud and Elnour [55] showed that ginger had a great ability to reduce body weight through increasing peroxisomal catalase level and HDL-c. Han et al. [57] reported that zingerone extracted from ginger could prevent the fat storage by increasing phrine-induced lipolysis in adipocytes. Body weight can be affected by many factors such as lifestyle, exercise, diet, and endocrine. So the nonsignificant effect of ginger reducing body weight may be caused by short duration in our quantitative analysis. Longer-term randomized controlled trials are thus needed to verify the effect of ginger on weight loss.

### 4.4. Limitations

Several possible limitations of the current review are worthy of comment. Firstly, although a broad search strategy was applied to minimize the publication bias, some language bias may exist; due to that this meta-analysis only included studies published in English or Chinese. In addition, the limitation of available literatures and total sample size restricted results validity. Moreover, due to the short duration of intervention in most studies, the possibility of detecting statistically significant change in BMI was limited. Therefore, better-designed, adequately powered, and longer-term RCTs are needed to explore the effects of ginger on T2DM and components of the MetS.

## 5. Conclusions

The systematic review and meta-analysis provide convincing evidence for the effects of ginger on glucose control, insulin sensitivity, and improvement of blood lipid profile. Based on the positive effects and negligible side effects, ginger may be a promising adjuvant therapy for T2DM and MetS. Further high-quality studies with larger sample sizes and longer duration of treatment are needed to examine these findings and evaluate the potential BMI lowering effect of ginger.

## Figures and Tables

**Table 1:** Summary of studies included in the systematic review.

First author (Ref number) (y) (country)	Number of ginger/control groups	Population characteristics	Experimental intervention/dosage	Control intervention/dosage	Follow-up duration	Outcomes	Study design
Alizadeh-Navaei, [21] (2008) (Iran)	45/40	Hyperlipidemia/53.6 ± 11 y^a^	Ginger capsule (3 g/d)	Lactose capsule (3 g/d)	45 days	Dec: TG, TC, LDL-c. Inc: HDL-c.	RCT

Andallu, [22] (2003) (India)	8/8	T2DM/men/weight (50–70 kg)/40–60 y^b^	Ginger capsule (3 g/d)	NR	30 days	Dec: FBG, TG, TC, LDL-c. Inc: HDL-c.	NR

Arablou, [23] (2014) (Iran)	33/30	T2DM/BMI (20–35)/HbA1c (7–10%)/30–70 y	Ginger capsule (1.6 g/d)	Wheat flour capsule (1.6 g/d)	12 weeks	Dec: FBG, HbA1c, INS, HOMA-IR, TG, TC. NC: LDL-c, HDL-c, BMI.	RCT

Mahluji, [24] (2013) (Iran)	28/30	T2DM (at least 2 years)/mean BMI (29.5)/38–65 y	Ginger tablet (2 g/d)	Corn starch tablet (2 g/d)	2 months	Dec: INS, HOMA-IR, TG, LDL-c. NC: FBG, HbA1c, TC, HDL-c, BMI.	RCT

Mozaffari-Khosravi, [25] (2014) (Iran)	40/41	T2DM (at least 10 years)/BMI < 40/30–70 y	Ginger capsule (3 g/d)	Cellulose microcrystalline capsule (3 g/d)	8 weeks	Dec: FBG, HbA1c. NC: INS, HOMA-IR, BMI.	RCT

Shidfar, [26] (2015) (Iran)	22/23	T2DM (at least 2 years)/BMI ≤ 30/HbA1c (6–8%)/20–60 y	Ginger capsule (3 g/d)	Lactose capsule (3 g/d)	3 months	Dec: FBG, HbA1c, INS, HOMA-IR.	RCT

Atashak, [27] (2011) (Iran)	8/8	Obese men/BMI ≥ 30/18–30 y	Ginger capsule (1 g/d)	Maltodextrin capsule (1 g/d)	10 weeks	NC: BMI, TG, TC, LDL-c, HDL-c.	RCT

Attari, [28] (2015) (Iran)	39/31	Obese women/BMI (30–40)/18–45 y	Ginger tablet (2 g/d)	Corn starch tablet (2 g/d)	12 weeks	Dec: TG. NC: FBG, TC, LDL-c, HDL-c.	RCT

Attari, [29] (2016) (Iran)	39/31	Obese women/BMI (30–40)/18–45 y	Ginger tablet (2 g/d)	Corn starch tablet (2 g/d)	12 weeks	Dec: INS, HOMA, BMI. NC: FBG.	RCT

Karimi, [30] (2015) (Iran)	10/10	Obese women (with breast neoplasms)/BMI (29.78 ± 3)/30–60 y	Ginger capsule (3 g/d)	Starch (4 g/d)	6 weeks	NC: FBG, INS, IR, TG, TC, LDL-c, HDL-c.	RCT

Imani, [31] (2015) (Iran)	18/18	CAPD (with hyperglycemia or dyslipidemia)/29–79 y	Ginger capsule (1 g/d)	Starch (1 g/d)	10 weeks	Dec: FBG.	RCT

Tabibi, [32] (2016) (Iran)	18/18	CAPD (with hyperglycemia or dyslipidemia)/29–79 y	Ginger capsule (1 g/d)	Starch (1 g/d)	10 weeks	Dec: TG. NC: TC, LDL-c, HDL-c.	RCT

T2DM: type 2 diabetes mellitus, CAPD: continuous ambulatory peritoneal dialysis, BMI: body mass index, y: year, g: gram, d: day. FBG: fasting blood glucose, HbA1c: glycosylated hemoglobin, INS: fasting insulin, HOMA-IR: homeostasis model assessment-insulin resistance index, TG: triglyceride, TC: total cholesterol, LDL-c: low density lipoprotein-cholesterol, HDL-c: high density lipoprotein-cholesterol. NC: not changed, Dec: deceased, Inc: increased, NR: not reported. RCT: randomized controlled trial. ^a^Range instead of mean ± standard deviation. ^b^Age at the follow-up.

## Supplementary Materials

Supplementary 1 eTable  1: search strategy in PubMed.

Supplementary 2 eTable  2: Cochrane risk of bias assessment of the studies included in the systematic review.

Supplementary 3 eFigure  1: funnel plots detailing publication bias in studies of the effect of ginger on glucose control and insulin sensitivity indexes; A for FBG, B for HbA1c, C for INS, and D for HOMA-IR.

Supplementary 4 eFigure  2: funnel plots detailing publication bias in studies of the effect of ginger on lipid indexes; A for TG, B for TC, C for LDL-c, and D for HDL-c.

Supplementary 5 eFigure  3: funnel plots detailing publication bias in studies of the effect of ginger on BMI.
