# Supplementary material for: Effects of Ginger (Zingiber officinale Roscoe) on Type 2 Diabetes Mellitus and Components of the Metabolic Syndrome: A Systematic Review and Meta-Analysis of Randomized Controlled Trials
Source: Evid Based Complement Alternat Med. 2018 Jan 9;2018:5692962. doi: 10.1155/2018/5692962 (PMC5818945; doi:10.1155/2018/5692962)
Supplement: Supplementary 1 — eTable 1: search strategy in PubMed. [file 5692962.f1.docx]

eTable 1- Search strategy in Pubmed

| Number | Search items |
| --- | --- |
| 1 | overweight [MH] OR overweight [tiab] OR obesity [tiab] OR obese [tiab] |
| 2 | Diabetes Mellitus, Type 2 [MH] OR diabet* [tiab] OR NIDDM [tiab] OR T2DM [tiab] OR hyperglycemi* [tiab] OR hyperglycaemi* [tiab] OR insulin resistance [tiab] OR hypoglycemia [tiab] OR blood glucose [tiab] OR FBS [tiab] OR FBG [tiab] |
| 3 | Dyslipidemias [MH] OR dyslipidemia? [tiab] or dyslipoproteinemia? [tiab] or Hyperlipidemia? [tiab] or Hyperlipoproteinemia? [tiab] or Hypercholesterolemia [tiab] or cholesterol [tiab] OR triglyceride [tiab] OR TG [tiab] OR high density lipoprotein [tiab] OR HDL [tiab] or low density lipoprotein [tiab] OR LDL [tiab] or Total cholesterol [tiab] OR TC [tiab] |
| 4 | coronary disease [MH] OR“Coronary Disease?” [tiab] OR “Coronary heart disease?” [tiab] OR “coronary artery disease” [tiab] OR CHD [tiab] |
| 5 | Hypertension [MH] OR Hypertension [tiab] or Blood Pressure? [tiab] |
| 6 | metabolic syndrome [MH] |
| 7 | #1 OR #2 OR #3 OR #4 OR #5 OR #6 |
| 8 | Ginger [MH] OR ginger* [tiab] OR Zingiber* [tiab] OR Zinziber* [tiab] |
| 9 | randomized [tiab] OR placebo [tiab] OR drug therapy [sh] OR randomly [tiab] OR trial [tiab] OR groups [tiab] |
| 10 | animals [mh] NOT humans [mh] |
| 11 | #9 NOT #10 |
| 12 | #7 AND #8 AND #11 |
