# Supplementary material for: Effects of Ginger (Zingiber officinale Roscoe) on Type 2 Diabetes Mellitus and Components of the Metabolic Syndrome: A Systematic Review and Meta-Analysis of Randomized Controlled Trials
Source: Evid Based Complement Alternat Med. 2018 Jan 9;2018:5692962. doi: 10.1155/2018/5692962 (PMC5818945; doi:10.1155/2018/5692962)
Supplement: Supplementary 2 — eTable 2: Cochrane risk of bias assessment of the studies included in the systematic review. [file 5692962.f2.docx]

| eTable 2 - Cochrane risk of bias assessment of the studies included in the systematic review. | | | | | | | |
| --- | --- | --- | --- | --- | --- | --- | --- |
| First author (y) | Random sequence generation | Allocation concealment | Blinding of participants and personnel | Blinding of outcome assessment | Incomplete outcome data | Selective reporting | Other bias |
| Alizadeh-Navaei 2008 | Unclear | Unclear | Low | Low | Unclear | Unclear | Unclear |
| Andallu 2003 | High | High | High | High | Unclear | High | Unclear |
| Arablou 2014 | Unclear | Unclear | Low | Low | Low | Unclear | Unclear |
| Mahluji 2013 | Unclear | Low | Low | Low | Low | Unclear | Low |
| Mozaffari-Khosravi 2014 | Low | Low | Low | Low | Low | Low | Unclear |
| Shidfar 2015 | Low | Low | Low | Low | Unclear | Unclear | Unclear |
| Atashak 2011 | Unclear | Unclear | Low | Low | Unclear | High | Unclear |
| Attari 2015 | Low | Low | Low | Low | Low | High | Unclear |
| Attari 2016 | Low | Low | Low | Low | Low | High | Unclear |
| Karimi 2015 | Unclear | High | High | High | Unclear | High | Unclear |
| Imani 2015 | Unclear | Unclear | Unclear | Unclear | Low | High | Unclear |
| Tabibi 2016 | Unclear | Unclear | Unclear | Unclear | Low | High | Unclear |
